# Supplementary material for: Complexome profiling of the Chlamydomonas psb28 mutant reveals TEF5 as an early PSII assembly factor
Source: Plant Cell. 2025 Mar 17;37(6):koaf055. doi: 10.1093/plcell/koaf055 (PMC12164590; doi:10.1093/plcell/koaf055)
Supplement: koaf055_Supplementary_Data [file koaf055_supplementary_data.zip › psb28_interactive.zip!/plots/intensity/qualityPlots/peptides/_Cre01.g028600.t1.2.html]

### Cre01.g028600.t1.2

Trivial name:   
SeqCoverage [median]: 15.50  
Mass: 26.49 kDa  
  
 

| Sequence | MissedCleavage | Mass [Da] | wtIntensity | muIntensity | Score |
| --- | --- | --- | --- | --- | --- |
| ITSSPAALK | 0 | 886.51 | 0.00 | 591.70 | 55.68 |
| LAALQGR | 0 | 727.43 | 0.00 | 427.22 | 72.00 |
| LAAQPGAQVVLAGR | 0 | 1349.78 | 0.00 | 397.35 | 61.65 |
| VIATAAETYGK | 0 | 1122.59 | 0.00 | 809.62 | 96.33 |
|  |  |  | 0.0 | 2225.9 |  |
